# Association of smart elderly care and quality of life among older adults: the mediating role of social support

**DOI:** 10.1186/s12877-024-05073-3

**Published:** 2024-05-29

**Authors:** Xi Chen, Miaoling Wu, Dongbo Wang, Jian Zhang, Bo Qu, Yaxin Zhu

**Affiliations:** 1 College of Health Management, China Medical University, No. 77 Puhe Road, Shenyang North New Area, Shenyang, 110122 Liaoning P.R. China; 2 Beijing Tongren Hospital Capital Medical University, No. 1 Dongjiaominxiang Dongcheng District, Beijing, 100730 China; 3 Institute for International Health Professions Education and Research, China Medical University, No. 77 Puhe Road, Shenyang North New Area, Shenyang, 110122 Liaoning P.R. China

**Keywords:** Quality of life, Smart elderly care, Healthy ageing, Community care, Institutional care

## Abstract

**Background:**

In the current context of ageing, the field of smart elderly care has gradually developed, contributing to the promotion of health among older adults. While the positive impact on health has been established, there is a scarcity of research examining its impact on the quality of life (QoL). This study aims to investigate the mediating role of social support in the relationship between smart elderly care and QoL among older adults.

**Methods:**

A total of 1313 older adults from Zhejiang Province, China, participated in the study. Questionnaires were used to collect data on participants’ basic demographic information, smart elderly care, social support, and QoL. The descriptive analyses of the demographic characteristics and correlation analyses of the three variables were calculated. Indirect effects were tested using bootstrapped confidence intervals (CI).

**Results:**

The analysis revealed a positive association between smart elderly care and social support (β = 0.42, *p* < 0.01), as well as a positive correlation between social support and QoL (β = 0.65, *p* < 0.01). Notably, social support emerged as an important independent mediator (effect size = 0.28, 95% bootstrap CI 0.24 to 0.32) in the relationship between smart elderly care and QoL.

**Conclusions:**

The results of this study underscore the importance of promoting the utilization of smart elderly care and improving multi-faceted social support for older adults, as these factors positively contribute to the overall QoL.

## Background

Population ageing stands out as one of the most significant global public health challenges in the 21st century. According to the United Nations, the world’s population age structure is undergoing profound changes due to rising life expectancy and declining fertility rates, with the proportion of older adults within the total population increasing rapidly [1]. The same is true for China, which is already one of the most rapidly ageing countries [2]. The complexity of ageing received increasing attention in recent years. The World Health Organization (WHO) has proposed the concept of ‘active ageing’, which focuses on optimizing the opportunities for health, participation, and security to enhance the quality of life (QoL) as individuals age [3]. WHO defines QoL as an individual’s perception of their position in life within the context of the culture and value systems they inhabit, relative to their goals, expectations, standards, and concerns [4]. Contemporary consensus among researchers underscores QoL among older adults as a multidimensional concept, including physical, psychological, and social dimensions of health [5]. Studying QoL among older adults is vital for gaining a comprehensive understanding of challenges existing in their daily lives, enabling the implementation of targeted strategies to promote active ageing [6]. QoL serves as an important indicator of the health status of the older population [7, 8]. In a survey of older Chinese individuals, nearly half reported experiencing chronic diseases [9]. Additionally, data from the American Association of Retired Persons showed that about 25% of older adults over the age of 70 suffered from a feeling of loneliness [10]. Enhancing the QoL of older population is imperative [11]. Therefore, it is highly important to explore the factors associated with QoL to develop interventions aimed at improving the well-being of older adults.

As the global ageing situation becomes increasingly severe, challenges such as the rising demand for elderly care and inadequate resources have surfaced. To address these issues, various ageing industries have emerged, with smart elderly care standing out as a rapidly growing sector expected to play a major role in enhancing elderly care quality and efficiency. Smart elderly care refers to the use of modern science and technology to support the life services and management of older adults in areas such as daily life, medical care, and health services, encompassing technologies like smart homes and e-health devices [12]. In recent years, the fourth scientific and technological revolution characterized by the Internet of Things, Big Data, and Cloud Computing has substantially promoted the development of smart elderly care. This industry is committed to providing various services that cater to the needs of older adults, including safety, independence, health, and efficient assistance [13]. Frisardi and Imbimbo found that the use of smart homes can monitor the activity and vital signs of older adults [14]. Additionally, a cross-sectional survey indicated that smart home solutions could promote or maintain the independence of older adults [15]. Furthermore, Pierleoni et al. found that a wearable fall detection system can provide timely medical assistance by identifying fall events in older adults through acceleration and orientation thresholds [16]. Despite the comprehensive nature of QoL, the impact of smart elderly care on QoL has been relatively unexplored [17]. Therefore, the purpose of this study is to analyze the effects and mechanisms of smart elderly care on QoL, aiming to provide valuable insight for enhancing the overall health of older adults.

Smart elderly care, encompassing technologies like smart homes, telehealth, and smart communication devices, has proven effective in promoting various aspects of health among older adults [18–21]. Research indicates that health monitoring within a smart home platform can facilitate remote monitoring of the home environment, important physiological signs, and the activities of older adults [18]. Sheeran et al. reported that telehealth monitoring technology not only provided depression care management but also led to an improvement in depression severity among older adults [19]. Moreover, Edwards et al. demonstrated that smart speakers provided companionship for older adults, contributing to the enhancement of their mental well-being [20]. Jeong et al. found that smartphones have the potential to help older adults maintain social connectedness and reduce loneliness [21]. Therefore, this study proposes Hypothesis 1: Smart elderly care can positively predict the QoL of older adults.

Social support represents the older adults’ belief in the potential assistance their networks may provide and the quantity and quality of that support [22]. Smart elderly care is dedicated to providing various supporting services for elderly care to meet their diverse needs [13]. Beyond offering necessary medical support by monitoring physiological indicators and providing medication reminders, smart elderly care also aims to establish a social connection network for older adults, thereby reducing social isolation through Internet information technology [23]. Costa et al. developed an application based on smart TV technology that delivers medical support to older adults, including medication reminders or telemedicine services [24]. For homebound older adults lacking social connections, telebehavioral activation is expected to alleviate loneliness and enhance social interaction [25]. Furthermore, smart homes assist older adults in maintaining social relationships, addressing the need for independent living [26]. In essence, smart elderly care provides different types of social support for older adults. Therefore, this study proposes Hypothesis 2: Smart elderly care can positively predict social support.

In a survey conducted by Sarla et al. [27] findings revealed that older adults with higher levels of social support tend to exhibit better health status. Empirical studies have consistently shown that social support not only promotes positive mental health but also mitigates negative psychological outcomes among older adults [28, 29]. Koelmel et al. [30] showed that social support from family members can reduce the level of depression. Additionally, support from peers and neighbors is also an important factor in improving the QoL for older adults [31]. Therefore, this study proposes Hypothesis 3: Social support can positively predict the QoL of older adults. Consequently, social support plays a mediating role in the relationship between smart elderly care and QoL.

Therefore, this study introduces Hypothesis 4: smart elderly care will directly predict the QoL of older adults, but also through the indirect path of social support.

## Materials and methods

### Ethics statement

Approval for this study was obtained from the ethics committee of China Medical University (2,019,048). Before the commencement of the study, participants were informed about the study’s purpose and assured that their privacy would be protected. Older adults voluntarily completed an anonymous self-administered questionnaire, and written informed consent was obtained from all participants.

### Participants

This cross-sectional study was conducted among older adults in Zhejiang Province from October to December 2020. Utilizing stratified random sampling, two districts and counties each in Hangzhou, Huzhou, and Jiaxing in northern Zhejiang Province were selected as the survey areas. Subsequently, five communities and three elderly care institutions were randomly selected in each district and county as survey sites, resulting in a total of 30 communities and 18 institutions. A face-to-face questionnaire survey method was employed to collect data. The survey included 1440 older adults, and after data collection, 1313 valid questionnaires were considered, resulting in an effective response rate of 91.2%.

### Measurements

The research questionnaire comprised sections on basic demographic information, QoL, and social support. From the literature review, we defined the concept of smart elderly care [12, 14, 21, 32]. It included categories such as (1) Smart home: encompassing smart door locks, security alarms, and monitoring, (2) Health monitoring: involving electronic blood pressure monitors and smartwatches; and (3) Communication: covering smartphones and internet platforms. Respondents were then asked if they had used these or other smart elderly care products in the past three months.

To assess the utilization of smart elderly care among older adults through questionnaires, the concept of smart elderly care was explained to participants.

#### Quality of life

In this study, the Short Form Health Survey (SF-12) was employed to measure QoL [33]. SF-12 measures eight domains which are divided into physical (functioning, role physical, pain, general health) and mental health (vitality, social functioning, role emotional, and mental health). Item scores were converted to a 100-point scale. The resulting score can be divided into two components: Physical Component Summary (PCS) and Mental Component Summary (MCS), which is obtained by averaging the four components of each domain. Higher scores indicate better QOL. This questionnaire has been validated for use in Chinese older adults [34]. In the current study, the Cronbach’s alpha value for this scale was 0.62, indicating acceptable reliability.

#### Social support

Social support among older adults was measured using the Medical Outcomes Study Social Support Survey (MOS-SSS) [35]. This scale, validated in the Chinese older population [36], comprises 20 items distributed across four dimensions: Tangible Support (TS, 4 items), Information and Emotional Support (IES, 8 items), Positive Social Interaction (PSI, 4 items), and Affective Support (AS, 3 items). A five-point Likert scale is used, with a total score of 19 ~ 95 points, with higher scores indicating better social support. In the current study, the Cronbach’s alpha value for this scale was 0.97, indicating good internal consistency.

### Data analysis

In this study, SPSS 25.0 was used for data input and organization. Descriptive statistics, including means and standard deviations or frequencies and percentages, were employed to represent the characteristics of study variables. The differences in QoL and social support by characteristics of the respondents were analyzed by t-test and ANOVA with LSD post hoc. The correlation between variables was explored through Chi-squared tests and Pearson correlation. Regression analysis was employed to verify the influence among variables. Amos 25.0 software was used to establish a smart elderly care model and further verify the serial mediation role of smart elderly care services and social support. The bias-corrected percentile bootstrap method (with 5000 samples under a 95% confidence interval) was used to test the mediation effect.

## Result

### Descriptive statistics and correlation analysis

The study surveyed a total of 1313 older adults, including 579 men (44.1%) and 734 women (55.9%), with an average age of (76.29 ± 9.29) years. Table 1 indicates that the QoL and social support scores of older adults in various age groups were significantly different (*P* < 0.001), with the highest scores in the 60–69 age group. The PCS and MCS scores of older adults in the community care model were significantly higher than those in institutional care (*P* < 0.001). The QoL scores of older adults who had used smart elderly care were significantly higher than those who had not (*P* < 0.001). Furthermore, the social support score of older adults who used smart elderly care in each dimension (*P* < 0.001) was significantly higher than that of older adults who did not use smart elderly care.

**Table 1:** Differences in the QoL and social support of older people under different basic conditions (*N* = 1313)

Variable		*N*	PCS (Mean ± SD)	MCS (Mean ± SD)	TS (Mean ± SD)	IES (Mean ± SD)	AS (Mean ± SD)	PSI (Mean ± SD)
Gender								
Male		579	44.91 ± 7.89	43.36 ± 8.85	65.35 ± 19.42	66.70 ± 17.97	67.25 ± 19.42	66.53 ± 18.88
Female		734	43.35 ± 8.40	43.48 ± 8.96	63.43 ± 20.11	65.37 ± 18.15	65.03 ± 19.69	64.73 ± 19.30
*T*			3.44***	-0.26	1.75	1.32	2.04*	1.70
Age								
60–69		364	47.53 ± 7.86	45.97 ± 8.60	70.20 ± 19.16	71.22 ± 17.90	72.44 ± 19.16	71.98 ± 18.81
70–79		409	44.90 ± 7.96^A^	44.35 ± 9.38^B^	66.80 ± 18.80^B^	67.99 ± 17.50^B^	68.15 ± 18.66^C^	67.20 ± 18.80^A^
80–89		437	41.61 ± 7.69^D, E^	41.24 ± 8.49^D, E^	59.02 ± 19.73^D, E^	61.14 ± 18.07^D, E^	50.46 ± 19.60^D, E^	60.26 ± 18.66^D, E^
≥ 90		103	38.57 ± 6.37^F, G, H^	40.08 ± 6.35^F, G^	55.73 ± 18.09^F, G^	59.96 ± 13.75^F, G^	58.38 ± 15.49^F, G^	58.35 ± 14.66^F, G^
*F*			58.05***	26.39***	31.74***	28.01***	33.96***	32.95***
Care model								
Institutional care		474	38.69 ± 5.68	39.11 ± 7.12	51.72 ± 16.01	55.96 ± 14.66	54.33 ± 15.67	54.22 ± 14.95
Community care		839	47.06 ± 7.88	45.87 ± 8.90	71.38 ± 18.20	71.61 ± 17.37	72.61 ± 18.48	71.91 ± 18.27
*t*			22.19***	15.04***	19.62***	16.56***	18.16***	17.96***
Smart elderly care								
No		488	39.69 ± 7.26	39.80 ± 8.59	52.42 ± 19.97	57.31 ± 17.73	56.12 ± 19.06	55.76 ± 18.30
Yes		825	46.61 ± 7.65	45.57 ± 8.39	71.30 ± 16.04	71.07 ± 16.25	71.86 ± 17.44	71.30 ± 17.17
*t*			16.36***	11.94***	17.77***	14.01***	14.92***	15.21***

PCS physical component summary, MCS mental component summary, TS tangible support, IES information and emotional support, AS affective support, SI positive social interaction, SD standard deviation, *t* value: Student’s t-test, *F* value: ANOVA, *p < 0.05, ***p < 0.001, Post hoc tests: A: significant difference from 60–69 group (*p* < 0.001), B: significant difference from 60–69 group (*p* < 0.05), C: significant difference from 60–69 group (*p* < 0.01), D: significant difference from 60–69 group (*p* < 0.001), E: significant difference from 70–79 group (*p* < 0.001), F: significant difference from 60–69 group (*p* < 0.001), G: significant difference from 70–79 group (*p* < 0.001), H: significant difference from 80–89 group (*p* < 0.001)

The chi-square test (Table 2) revealed there were statistically significant differences in the use of smart elderly care services among older adults in different age groups (χ^2^ = 32.07, *P* < 0.001). The proportion of older adults in the younger age group using smart elderly care services is higher than others. Additionally, there were statistically significant differences in the use of smart elderly care services (χ^2^ = 149.49, *P* < 0.001) among older adults with different care models. Specifically, 41.1% of older adults in the institutional care model had used smart care, a proportion lower than that observed in the community care model, where 75.1% of individuals had utilized smart elderly care services. There was no significant difference of older adults with different genders.

**Table 2:** Differences in smart elderly care services for older people under different basic conditions (*N* = 1313)

Variable		Smart elderly care		χ^2^	
		Yes	No		
Gender					
Male		379(65.5%)	200(34.5%)	3.06	
Female		446(60.8%)	288(39.2%)		
Age					
60–69		251(69.0%)	113(31.0%)	32.07^***^	
70–79		281(68.7%)	128(31.3%)		
80–89		245(56.1%)	192(43.9%)		
≥ 90		48(46.6%)	55(53.4%)		
Care model					
Institutional care		195(41.1%)	279(58.9%)	149.49^***^	
Community care		630(75.1%)	209(24.9%)		

***p < 0.001

Pearson correlation analysis found that the scores of PCS and MCS were positively correlated with the score of social support (0.33 ≤ *r* ≤ 0.95, *P* < 0.001), all of which were statistically significant, as shown in Table 3.

**Table 3:** Correlation analysis of QoL and social support for older adults (*N* = 1313)

Variable	Score (Mean ± SD)	PCS	MCS	TS	IES	AS	PSI	MOS-SSS-C
PCS	44.04 ± 8.21	1.00	0.33*	0.44*	0.40*	0.44*	0.45*	0.46*
MCS	43.43 ± 8.91	0.33*	1.00	0.45*	0.41*	0.44*	0.44*	0.47*
TS	64.28 ± 19.82	0.44*	0.45*	1.00	0.77*	0.79*	0.79*	0.90*
IES	65.96 ± 18.08	0.40*	0.41*	0.77*	1.00	0.87*	0.88*	0.94*
AS	66.01 ± 19.59	0.44*	0.44*	0.79*	0.87*	1.00	0.88*	0.95*
PSI	65.52 ± 19.13	0.45*	0.44*	0.79*	0.88*	0.88*	1.00	0.95*
MOS-SSS-C	65.44 ± 17.90	0.46*	0.47*	0.90*	0.94*	0.95*	0.95*	1.00

PCS physical component summary, MCS mental component summary, TS tangible support, IES information and emotional support, AS affective support, PSI positive social interaction, MOS-SSS-C medical outcomes study social support survey, SD standard deviation, *p < 0.05

### Mediation analyses

The regression analysis were adjusted for age and gender which are associated with QoL. The results presented in Table 4 shows that community care (β = 0.26, *P* < 0.001), the use of smart elderly care (β = 0.21, *P* < 0.001), and social support (β = 0.21, *P* < 0.001) all significantly and positively predicted the PCS score in older adults. Furthermore, the same table reveals that community care (β = 0.14, *P* < 0.001), the use of smart elderly care (β = 0.12, *P* < 0.001), and social support (β = 0.33, *P* < 0.001) all significantly and positively predicted the MCS score in older adults.

**Table 4:** Regression analysis of factors associated with QoL among older adults (*N* = 1313)

Predictor		PCS		MCS	
		β	t	β	t
Care model	Institutional care	-	-	-	-
	Community care	0.26	9.54***	0.14	4.67***
Smart elderly care	No	-	-	-	-
	Yes	0.21	8.39***	0.12	4.28***
MOS-SSS-C		0.21	7.66***	0.33	11.52***
*R²*		0.36		0.26	
*F*		105.56***		65.04***	

QoL quality of life, PCS physical component summary, MCS mental component summary, MOS-SSS-C medical outcomes study social support survey, ***p < 0.001

Using hierarchical regression analysis, a hypothesis model was established to explore the relationships among smart elderly care, social support, and QoL in older adults. The structural equation model was constructed based on this hypothesis to further demonstrate the mediating role of social support. In this study, the model fitting index meets the adaptation criteria, suggesting that the established model can be considered reasonable.

**Fig. 1:**
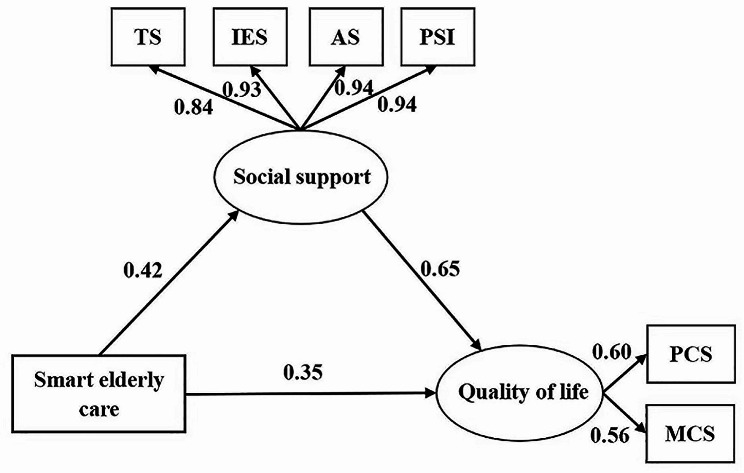
Path diagram of structural equation model between smart elderly care, social support, and QoL

The results of structural equation modeling (see Fig. 1) showed that compared with older adults who had not used smart elderly care, the utilization of smart elderly care could positively affect their QoL (β = 0.35, *P* < 0.01). In addition to direct effects, smart elderly care could also have a positive effect on social support (β = 0.42, *P* < 0.01), which subsequently positively predicts QoL (β = 0.65, *P* < 0.01). These results suggest that social support plays a mediating role in the relationship between smart elderly care and QoL.

Finally, the mediation effect is tested using the bootstrap method (see Table 5). The results indicate that both the direct and indirect effects of smart elderly care on QoL were statistically significant. This suggests that social support serves as a mediator and has a significant impact on the relationship between smart elderly care and QoL. The direct effect was 0.350, while the 95% CI was [0.273, 0.430], accounting for 55.82% of the total effect. The indirect effect was 0.277, while the 95% CI was [0.236, 0.317], accounting for 45.18% of the total effect.

**Table 5:** Mediating paths between smart elderly care and QoL

Route	Effect Size	Boot SE	Bootstrap 95% CI		Relative effect
			Boot LLCI	Boot ULCI	
SEC → QoL	0.350	0.040	0.273	0.430	55.82%
SEC → SP → QoL	0.277	0.020	0.236	0.317	45.18%
Total effect	0.627	0.038	0.553	0.704	100%

SEC smart elderly care, SP social support, QoL quality of life, SE Standard error, CI confidence interval, LL lower limit, UL upper limit

## Discussion

In this study, we observed a positive impact of smart elderly care on the QoL among older adults. Those who utilized smart elderly care reported higher QoL compared to those who did not, aligning with the hypothesis (H1) of this study. This finding resonates with a survey conducted in Australia, where smart home technology significantly supported older individual’s overall self-perceived QoL, particularly in terms of life satisfaction and future security [17]. When Pierleoni et al. [16] investigated smart wearable devices, they revealed their potential to monitor activities of daily living and accidental falls of older adults. In addition, communication devices such as smartphones can enhance social connections and effectively prevent the occurrence of negative emotions such as depression and loneliness in older adults [21]. Collectively, these findings suggest that smart elderly care could provide a feasible framework for improving the QoL of older adults across physiological, psychological, social, and other aspects. However, our study found that 37.2% of older adults have never utilized smart elderly care. According to previous studies, one of the possible reasons for this phenomenon may be the digital divide, where older adults have limited acceptance of digital technology, resulting in their lower willingness to choose smart elderly care [37–39]. Therefore, public institutions should strengthen the publicity and guidance on using digital technology for the elderly [38].

In addition, the study revealed a positive correlation between the level of social support in older adults and their QoL, which is consistent with the hypothesis (H3) of this study and similar to the findings of Sarla et al. [27] This observation is in accordance with the buffering model [40], which suggests that social support functions as a buffer or compensation for the negative effects of stress on health outcomes. Additionally, social support can increase the likelihood that individuals will adopt and maintain healthy behaviors [41]. Higher social support levels also imply increased social contact with others and build healthier social networks, helping older adults avoid negative emotions [28]. However, the lowest scores were assigned for tangible support in this study, indicating that older adults might be receiving financial support or insufficient practical assistance from society and family [42]. This is followed by positive social interactions, suggesting that older adults may feel less socially connected in their lives [43]. To address these challenges, policymakers should focus on the financial benefits of older adults, for example, by increasing pension coverage and amounts, and providing additional funding and support for those in need [44]. In addition, communities should be encouraged to establish recreational facilities and develop social activities for older adults to promote positive social interactions.

Furthermore, the study reveals that smart elderly care not only directly affects QoL but also indirectly affects QoL by positively influencing social support, which is consistent with the hypothesis (H4) of this study. In essence, smart elderly care aims to gather and provide various supports for older adults more intelligently and conveniently [45], to improve the QoL and happiness [21]. Smart wearable devices, for example, can monitor the physiological indicators of older adults in real time. In case of an issue, these devices can seek medical help through online consultation platforms [16], offering a certain degree of medical support. In addition, smart information devices can ensure that older adults can communicate with family and friends without barriers, access various information in society, and consequently receive affectionate support and positive social interaction [46]. Therefore, the utilization of smart elderly care correlates with higher social support levels among older adults, confirming the mediating effect of social support in smart elderly care and QoL.

Finally, the study uncovered that the proportion of older adults in the community care model using smart elderly care is higher than those in institutional care. Additionally, the QoL of older adults in community care is also higher, which is similar to the results of previous studies [47, 48]. This is mainly due to the distinctive nursing characteristics and environmental differences between the two care models. In the community care model, older adults may be inclined to use smart home and wearable monitoring devices to seek medical assistance while ensuring safety, and to help compensate for the potential lack of professional care [45]. On the other hand, in institutional care, older adults might favor using smartphones and intelligent online communication platforms. Even in relatively closed nursing homes, these technologies facilitate communication with family and friends without barriers, enhancing social connections [21]. Moving forward, it is imperative for the government to undertake targeted smart elderly care publicity campaigns tailored for older adults with different care models, and recommend products that meet their needs.

### Limitations

First, participants in this study were exclusively from one province in China, potentially limiting the generalizability of findings to the broader older population. Future studies involving diverse contexts are necessary for the replicability of results. Second, the cross-sectional design employed in this study cannot infer causal relationships between variables. Future longitudinal studies are necessary to verify causality. In addition, the study acknowledges the possibility of recall bias, which could affect the results. Thirdly, future research should explore what kind of smart elderly care are needed for older adults with different characteristics, and then study the impact on QoL. Finally, since the scope and concept of smart elderly care have not yet been established in the world, there may be limitations in the assessment of smart elderly care for older adults by using questions as a scale in this study. Future studies should further generalize this concept and use standardized questionnaires to measure this variable.

## Conclusions

In summary, this study analyzes the influence mechanism of smart elderly care on QoL by focusing on the mediation role of social support in older adults. Specifically, utilizing smart elderly care not only strengthens the social connections of older adults, but also ensures their health and safety, improves the level of social support, and contributes to their QoL. Our results support the need to pay attention to these two influencing factors in the process of healthy elderly care for older adults. Governments are encouraged to develop personalized strategies for older adults in different care models, promote increased usage of smart elderly care, and offer necessary guidance and diverse social support to achieve the goal of healthy ageing.

## Data Availability

The dataset used and analysed during the current study are available from the corresponding author on reasonable request.

## Abbreviations

QoL: Quality of Life
CI: Confidence Intervals
WHO: World Health Organization
SF-12: the Short Form Health Survey
PCS: Physical Component Summary
MCS: Mental Component Summary
MOS-SSS: Medical Outcomes Study Social Support Survey
TS: Tangible Support
IES: Information and Emotional Support
PSI: Positive Social Interaction
AS: Affective Support
